# Spatio-Temporal Analysis of Smear-Positive Tuberculosis in the Sidama Zone, Southern Ethiopia

**DOI:** 10.1371/journal.pone.0126369

**Published:** 2015-06-01

**Authors:** Mesay Hailu Dangisso, Daniel Gemechu Datiko, Bernt Lindtjørn

**Affiliations:** 1 Center for International Health, Faculty of Medicine and Dentistry, University of Bergen, Bergen, Norway; 2 Sidama Zone Health Department, Hawassa, Ethiopia; 3 Hawassa University, Hawassa, Ethiopia; 4 Liverpool School of Tropical Medicine, Pembroke Place, Liverpool, L3 5QA, United Kingdom; University of Tennessee, UNITED STATES

## Abstract

**Background:**

Tuberculosis (TB) is a disease of public health concern, with a varying distribution across settings depending on socio-economic status, HIV burden, availability and performance of the health system. Ethiopia is a country with a high burden of TB, with regional variations in TB case notification rates (CNRs). However, TB program reports are often compiled and reported at higher administrative units that do not show the burden at lower units, so there is limited information about the spatial distribution of the disease. We therefore aim to assess the spatial distribution and presence of the spatio-temporal clustering of the disease in different geographic settings over 10 years in the Sidama Zone in southern Ethiopia.

**Methods:**

A retrospective space–time and spatial analysis were carried out at the kebele level (the lowest administrative unit within a district) to identify spatial and space-time clusters of smear-positive pulmonary TB (PTB). Scan statistics, Global Moran’s *I*, and Getis and Ordi (Gi*) statistics were all used to help analyze the spatial distribution and clusters of the disease across settings.

**Results:**

A total of 22,545 smear-positive PTB cases notified over 10 years were used for spatial analysis. In a purely spatial analysis, we identified the most likely cluster of smear-positive PTB in 192 kebeles in eight districts (RR= 2, p<0.001), with 12,155 observed and 8,668 expected cases. The Gi* statistic also identified the clusters in the same areas, and the spatial clusters showed stability in most areas in each year during the study period. The space-time analysis also detected the most likely cluster in 193 kebeles in the same eight districts (RR= 1.92, p<0.001), with 7,584 observed and 4,738 expected cases in 2003-2012.

**Conclusion:**

The study found variations in CNRs and significant spatio-temporal clusters of smear-positive PTB in the Sidama Zone. The findings can be used to guide TB control programs to devise effective TB control strategies for the geographic areas characterized by the highest CNRs. Further studies are required to understand the factors associated with clustering based on individual level locations and investigation of cases.

## Introduction

Tuberculosis (TB) is an infectious disease affecting and claiming the lives of millions, with developing countries being hit the worst [1]. The magnitude of the problem varies across settings, possibly due to unfavorable socio-economic conditions, overcrowding, poverty, poor access to health services, socio-cultural barriers and HIV infection [1–6].

Increasing evidence about disease distribution is being generated using Geographic Information Systems (GIS) and scan statistics to analyze and detect spatial and spatio-temporal variations and clustering of diseases [7]. Various studies reported spatial [8–13] and spatio-temporal clustering [12, 14–16] of TB, thereby generating important information about the distribution of the disease and its transmission pattern, risk factors for the disease and the evaluation of intervention efforts [11–21]. However, most studies were conducted in urban settings over a short period of time, which make them deficient in detecting the pattern of the disease distribution in predominantly rural areas.

TB Reports from Ethiopia show variations in the trends and case notification rates by region [22], although little is known whether the variations are due to the spatial and spatio-temporal pattern of the disease. Moreover, the reports are based on basic management units (BMU). The BMU reports may include cases outside of the administrative catchment or miss cases from their catchment enrolled in neighboring health facilities, which could cause over- or underreporting. The reason for this is because patients could cross the administrative boundaries for seeking health services due to access, quality of care and preference of the patients. The national TB prevalence survey of Ethiopia reported a lower smear-positive TB prevalence (108 per 100,000 people) than WHO estimates [23]; however, the report did not show the spatial distribution and burden of the disease within- and between the lower administrative units.

Consequently, the national TB program implements similar interventions across settings regardless of the burden of the disease in the community, which could be due to a lack of evidence on the distribution pattern of the disease in different settings. Furthermore, information about the spatial distribution of the disease is limited, with the exception a single publication reporting a spatio-temporal variation in the northern part of the country [16]. Understanding the spatial pattern and spatio-temporal variations of the disease in wider geographic settings, including urban-rural areas, may help policy and decision-making in resource-constrained settings such as in Ethiopia. As a result, we aim to assess the spatial distribution and look for the spatio-temporal clustering of the disease over the past 10 years in the Sidama Zone in southern Ethiopia.

## Methods

### Study area and setting

**Fig 1:**
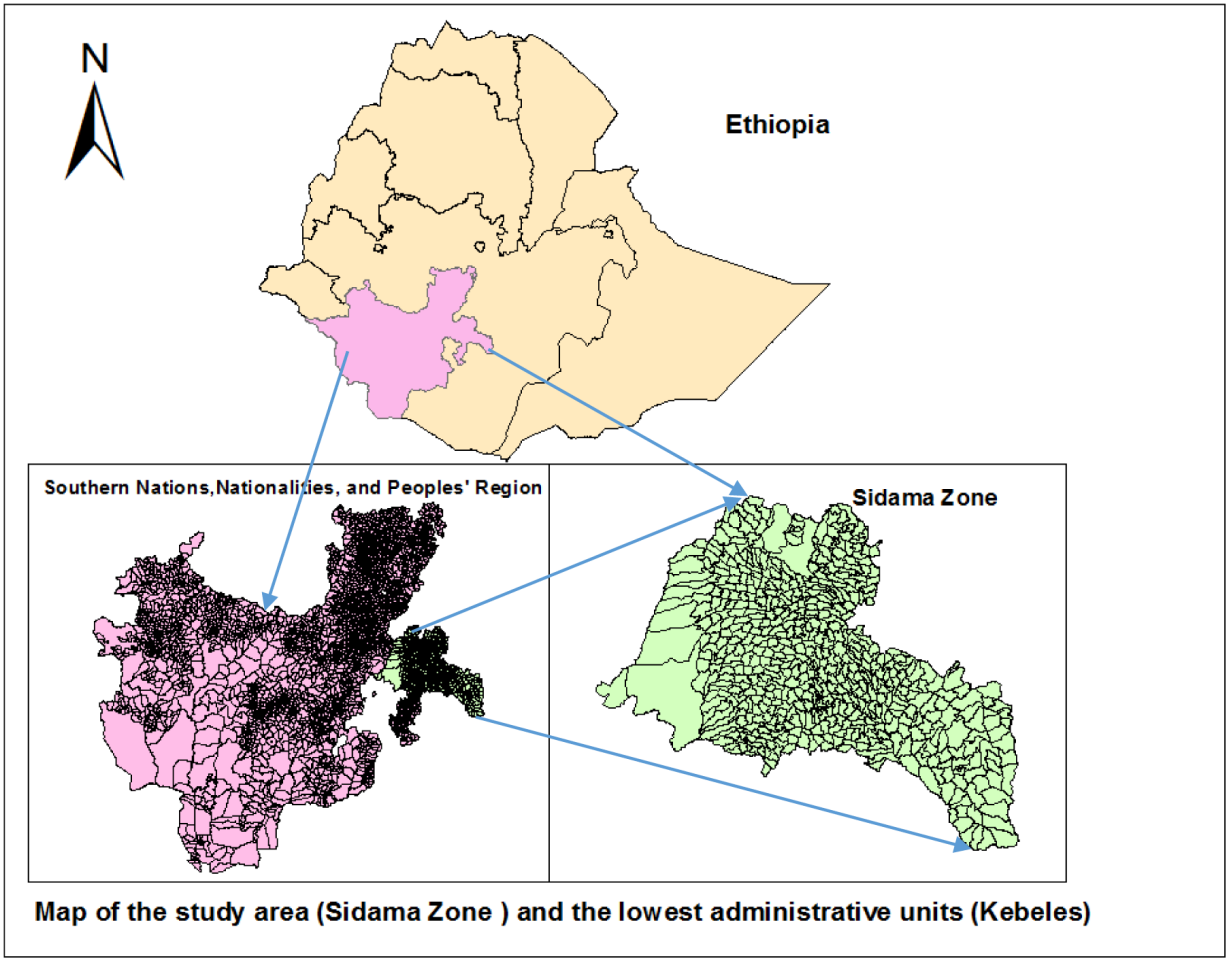
Map of the study area (Sidama Zone) and the lowest administrative units (kebeles).

The study was conducted in the Sidama Zone in southern Ethiopia, which is located between 6°14’ and 7°18’ N and 37°92’and 39°14’ E. The Zone is divided into 19 districts and two town administrations with a population of over 3.4 million and covers a geographic area of 6,982 square kilometers [24], while 92% of the population live in rural areas (Fig 1). There were 563 kebeles, which are the smallest administrative units within districts, with a population of 5,000 on average. In the study area, there were 39 urban and 524 rural kebeles.

### Data collection and analysis

Data were collected from August 2012 to February 2013. We collected the data from unit TB registers from all health facilities that provided Directly Observed Treatment Short-term (DOTS) services from 2003 to 2012, and matched individual cases to their place of residence using codes given by the Central Statistical Agency of Ethiopia (CSA). An address with similar names, but from other locations, was also identified and linked to their true address using location codes. The data collection was carried out using a semi-structured pretested questionnaire by university graduates after four days of practical training. The data were double entered and checked by the principal investigator (PI) and health management information system (HMIS) experts. In addition, the data were checked by year, district and health facilities against unit TB registers for consistency and completeness throughout the entire data collection process.

The data were entered into Microsoft Access, and the analysis was done using SPSS 20. The number of cases and patient information entered in each year were checked page-by-page and by year of treatment with the information in the TB registry. We carried out the exploratory analysis, looked for errors and corrected them. The corrected errors were a duplication of cases, or incomplete or missing information, while smear-positive PTB CNRs were computed for each district and kebele. We obtained CNRs by dividing the number of TB cases reported by health facilities over the population of a given year and multiplied by 100,000 to obtain the CNRs [1], and we used the total population of each kebele and district to calculate the respective CNRs of smear-positive PTB cases. Patient records or information was anonymized and de-identified prior to analysis.

Kebele centroids were used to represent a geographically weighted central location as coordinates. We also prepared an attribute table containing the population for each kebele, the number of cases, the case notification rate and the coordinates, and joined the variables of interest to ArcGIS 10.1. The coordinates’ projection was defined using the World Geodetic System (WGS) 1984, Universal Transverse Mercator (UTM) Zone 37°N. All TB cases were geo-coded and matched to the kebele level layers of polygon and point using ArcGIS 10.1. The mean and each year’s CNRs of TB in the districts and kebeles were computed. Spatial empirical Bayes smoothing (SEBS) was also applied in geographic data analysis tools (GeoDa) in order to overcome a variance instability in small areas, which is due to differences in population size, as well as few cases of disease in some areas [25].

In using the SEBS technique, the *prior* distribution to correct for the variance in instability is localized and based on a locally varying reference mean and variance. We used the number of smear-positive PTB cases as an event (numerator) and population for each location as a base (denominator) variable. A queen weights matrix (which defines the location’s neighbors as those with either a shared border or vertex) was used for spatial weights [25]. We used box plots as well as a comparison of raw and smoothed rates to help assess the sensitivity of the smoothing. Furthermore, we mapped the annualized and the smoothed rates [7] to explore the pattern of the disease distribution.

### Spatial autocorrelation analysis

We applied the Global Moran’s *I* statistics in ArcGIS 10.1 to help investigate the spatial autocorrelation and distribution pattern of smear-positive PTB in the study area. We also employed local Gi* statistics to examine the local level clusters and to determine the locations of clusters or hot spots. The Gi* statistics carry out the spatial analysis by looking at each feature within the context of a neighboring feature. The local sum for a feature and its neighbors is proportionally compared to the sum of all features. When the local sum is significantly different from the expected sum, and the difference is too large to be the result of random chance, a statistically significant Z score results [7, 26]. We used the mean rate of smear-positive PTB as the input field.

The equation for the Gi*statistics is [26]:

$$Gi*=\frac{\sum_{j=1}^{n}w_{i,j}x_{j}-\bar{X}\sum_{j}^{n}w_{i,j}}{S\sqrt{\frac{\left[n\sum_{j=1}^{n}w_{i}{}^{2}{,}_{j}-{\left(\sum_{j=1}^{n}w_{i,j}\right)}^{2}\right]}{n-1}}}$$

where *x*_*j*_ the attribute value (CNR) for feature *i*, *w*_*i*,*j*_ is the spatial weight, thus explaining the closeness between features *i* and *j*, *n*, which is equal to the total number of features and: $\bar{X}=\frac{\sum_{j=1}^{n}x_{j}}{n}$, and $S=\sqrt{\frac{\sum_{j=1}^{}x_{i}{}^{2}}{n}-{\left(\bar{X}\right)}^{2}}$. Therefore, the G_i_* statistic is a *Z*-score.

The computed value of Gi* ≥ 1.96 and a P-value of < 0.05 were both considered to be a statistically significant high rate.

#### Spatial analysis

A Kulldroff’s scan statistic (SaTScan 9.2) [27] was used for spatial and space-time analysis. Kulldroff’s scan statistics are a widely used tool for spatial and space-time cluster analysis for diseases in different settings [28, 29]. The scan statistics carry out a cluster analysis and detect cluster size and locations, compute the relative risk (RR) and provide a P-value using Monte Carlo Simulation. We used the number of cases (the aggregated data of cases of smear-positive PTB at the kebele level), population and coordinates as input files, as well as the discrete Poisson model, with the assumption that the number of cases at each location was Poisson distributed with a known population at risk. Scan circles of various sizes, including the default setting in scan statistics, was used to identify the most likely spatial clusters of smear-positive PTB. For maximum spatial cluster size, the upper limit, which is 50% of the population at risk, was used. The likelihood ratio was calculated to measure a relative risk [27], and the most likely and secondary clusters were identified and reported when a P-value was less than 0.05. The results of the analyses were presented in tables and on the maps to depict the locations where unusually high rates of the disease have occurred.

### Space-time analysis

The space-time scan statistic method applies a cylindrical window, in which the circular geographic base is corresponding to the space and height to time for potential clusters [27]. It assumes that the RR of smear-positive PTB was the same within the window compared to the outside. The Poisson probability model was used, in which the number of events in areas is Poisson-distributed according to a known population at risk [27]. The geographic size of the window was limited to half the expected number of cases, and the time was limited to the total time period. The test of significance was obtained from comparing the likelihood ratio test against a null distribution computed from a Monte Carlo Simulation. The number of permutations was set to 999, and P<0.05 was considered to be statistically significant. In 2010, an intensive case finding campaign was conducted in nine districts of the study area, and since 2011 a community-based active case finding intervention has been implemented in all districts in the study area to increase TB case notification. Thus, we carried out the space-time analysis for the period from 2003–2012 and sub-time-phases from 2009–2010 and 2011–2012 to help investigate the transmission pattern and a presence of recent space-time clusters of the disease.

### Ethical clearance

We obtained ethical approval and clearance from the ethical review committee of the Public Health Research and Technology Transfer Support Process at the Regional Health Bureau of southern Ethiopia. We also obtained a letter of support from the Sidama Zone Department of Health to obtain information from all districts and health facilities. Personal identifiers of the cases were coded prior to analysis and medical records were kept in a secure place to help maintain the confidentiality of the clinical information of cases.

## Results

A total of 37,333 cases were diagnosed and treated during the period from 2003 to 2012. Of these, 37,070 (99.3%) cases were from the study area, whereas 263 cases were from the neighboring areas. Most cases 22,545 (61%) were smear-positive PTB, while 7,996 (22%) were smear-negative and 6,464 (17%) were extra pulmonary TB. We used a total of 22,545 smear-positive PTB cases for spatial analysis over 10 years. The mean age (SD) of smear-positive PTB cases was 29 (SD = 14) years, and of the 22,545 smear-positive PTB cases, 10,296 (46%) were women and 12,240 (54%) were men, with a male to female ratio of 1:1.2. Ninety-five percent (21,302 cases) of the cases were new, and 5% (1,190 cases) were retreatment cases. Fifty eight percent of the cases were from seven districts: Boricha, Dale, Shebedino, Wondo Genet, Chuko, Aleta wondo and Bensa, with these districts constituting 48% of the study area population (Table 1). Urban areas account for 11% (2,448 cases), whereas the urban population was only 8% of the total population of the study area. Ninety-seven percent (21,793 cases) of the cases had a kebele address, with the exception of 3% (752 cases) with no kebele address, who were excluded from the spatial analysis.

**Table 1:** Number of smear-positive pulmonary tuberculosis cases notified in the Sidama Zone in southern Ethiopia, 2003–2012.

Districts	2003 N (%)	2004 N (%)	2005 N (%)	2006 N (%)	2007 N (%)	2008 N (%)	2009 N (%)	2010 N (%)	2011 N (%)	2012 N (%)	Total* N (%)
Rural districts											
Shebedino	172 (13)	140 (9)	95 (6)	112 (8)	150 (9)	169 (7)	183 (8)	154 (7)	342 (8)	345 (9)	1,862 (8)
Hawassa Zuria	63 (5)	80 (5)	63 (4)	80 (6)	82 (5)	130 (5)	73 (3)	104 (5)	165 (4)	135 (4)	975 (4)
Arbegona	8 (1)	7 (0.4)	6 (0.4)	1 (0.1)	12 (1)	63 (3)	87 (4)	47 (2)	132 (3)	89 (2)	452 (2)
Dale	168 (12)	135 (9)	144 (9)	104 (7)	227 (13)	263 (11)	294 (13)	252 (12)	445 (11)	388 (10)	2,420 (11)
Aleta Wondo	73 (5)	135 (9)	127 (8)	137 (10)	149 (9)	181 (7)	178 (8)	147 (7)	246 (6)	232 (6)	1,605 (7)
Dara	12 (1)	33 (2)	116 (7)	62 (4)	44 (3)	122 (5)	53 (2)	116 (5)	134 (3)	104 (3)	796 (4)
Hula	53 (4)	49 (3)	79 (5)	57 (4)	81 (5)	74 (3)	84 (4)	135 (6)	158 (4)	165 (4)	935 (4)
Bensa	56 (4)	87 (5)	139 (9)	85 (6)	109 (6)	142 (6)	137 (6)	150 (7)	240 (6)	243 (6)	1,388 (6)
Aroresa	6 (0.4)	32 (2)	59 (4)	37 (3)	123 (7)	200 (8)	82 (4)	109 (5)	154 (4)	255 (7)	1,057 (5)
Boricha	254 (19)	267 (17)	168 (11)	206 (14)	214 (12)	407 (16)	262 (11)	162 (8)	425 (11)	293 (8)	2,658 (12)
Gorche	22 (2)	14 (1)	0	1 (0.1)	8 (0.5)	23 (1)	41 (2)	41 (2)	133 (3)	93 (2)	376 (2)
Malga	29 (2)	44 (3)	33 (2)	31 (2)	34 (2)	55 (2)	42 (2)	41 (2)	101 (2)	95 (2)	505 (2)
Wonsho	40 (3)	29 (2)	37 (2)	33 (2)	45 (3)	30 (1)	27 (1)	39 (2)	153 (4)	140 (4)	573 (3)
Loka Abaya	48 (4)	31 (2)	28 (2)	28 (2)	24 (1)	66 (3)	52 (2)	48 (2)	200 (5)	126 (3)	651 (3)
Chire	0	7 (0.4)	16 (1)	30 (2)	31 (2)	47 (2)	90 (4)	54 (3)	130 (3)	317 (8)	722 (3)
Bursa	35 (3)	27 (2)	49 (3)	44 (3)	78 (5)	55 (2)	78 (3)	62 (3)	104 (3)	109 (3)	641 (3)
Chuko	130 (10)	147 (9)	120 (8)	128 (9)	120 (7)	178 (7)	204 (9)	165 (8)	243 (6)	225 (6)	1,660 (7)
Bona Zuriya	23 (2)	16 (1)	66 (4)	47 (3)	9 (1)	47 (2)	60 (3)	99 (5)	165 (4)	109 (3)	641 (3)
Wondo Genet	97 (7)	182 (12)	122 (8)	97 (7)	108 (6)	137 (5)	137 (6)	114 (5)	301 (7)	299 (8)	1,594 (7)
Town administrations											
Yirgalem town	58 (4)	56 (4)	49 (3)	59 (4)	34 (2)	66 (3)	76 (3)	68 (3)	59 (1)	49 (1)	574 (3)
Aleta town	11 (1)	68 (4)	58 (4)	55 (4)	52 (3)	47 (2)	46 (2)	49 (2)	26 (1)	40 (1)	452 (2)
Sidama Zone	1,358	1,586	1,574	1,434	1,734	2,502	2,286	2,156	4,056	3,851	22,537

*For eight cases, year of treatment was not mentioned.

### Spatial distribution of smear-positive PTB at the district- and kebele level

**Fig 2:**
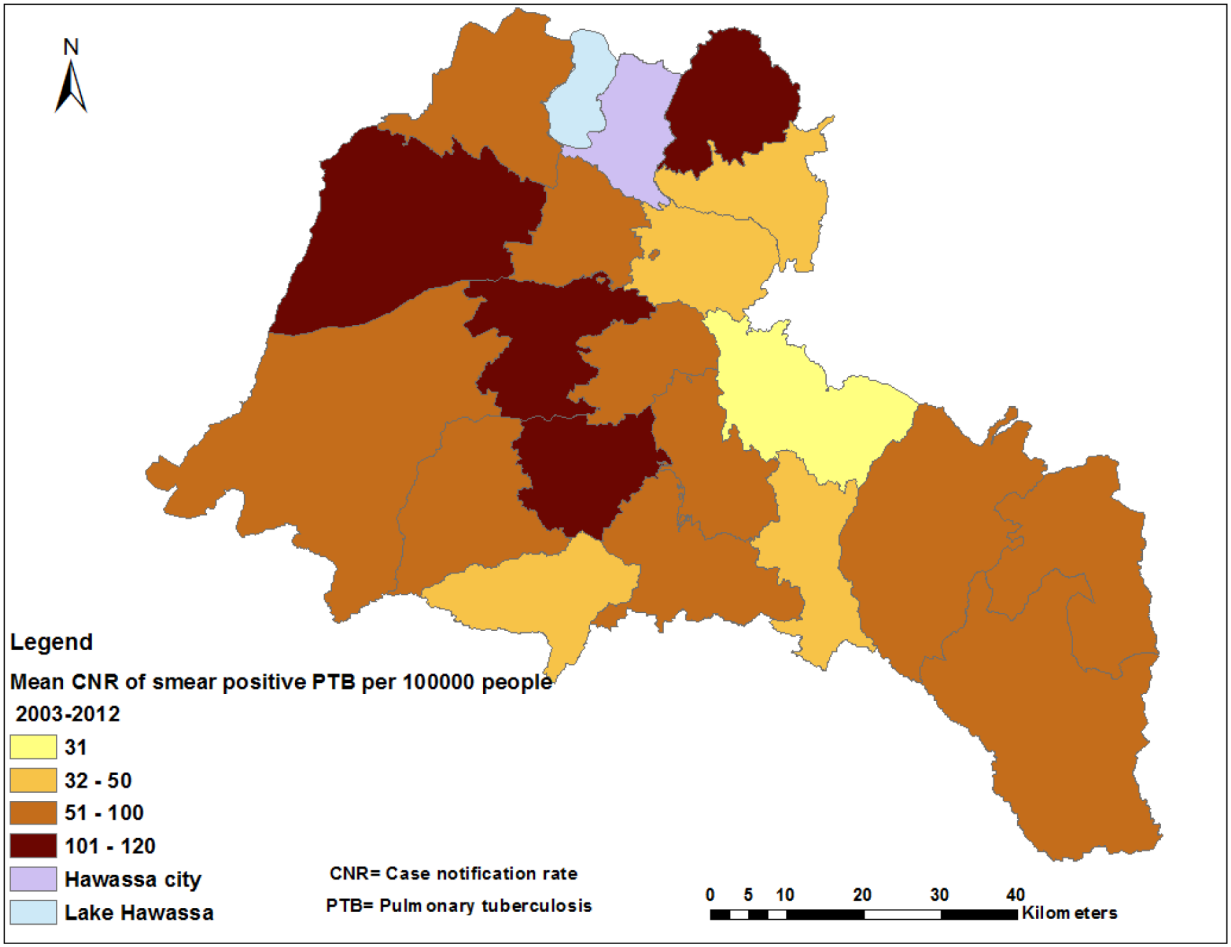
Spatial distribution of smear-positive pulmonary tuberculosis by district in the Sidama Zone in southern Ethiopia, 2003–2012.

**Fig 3:**
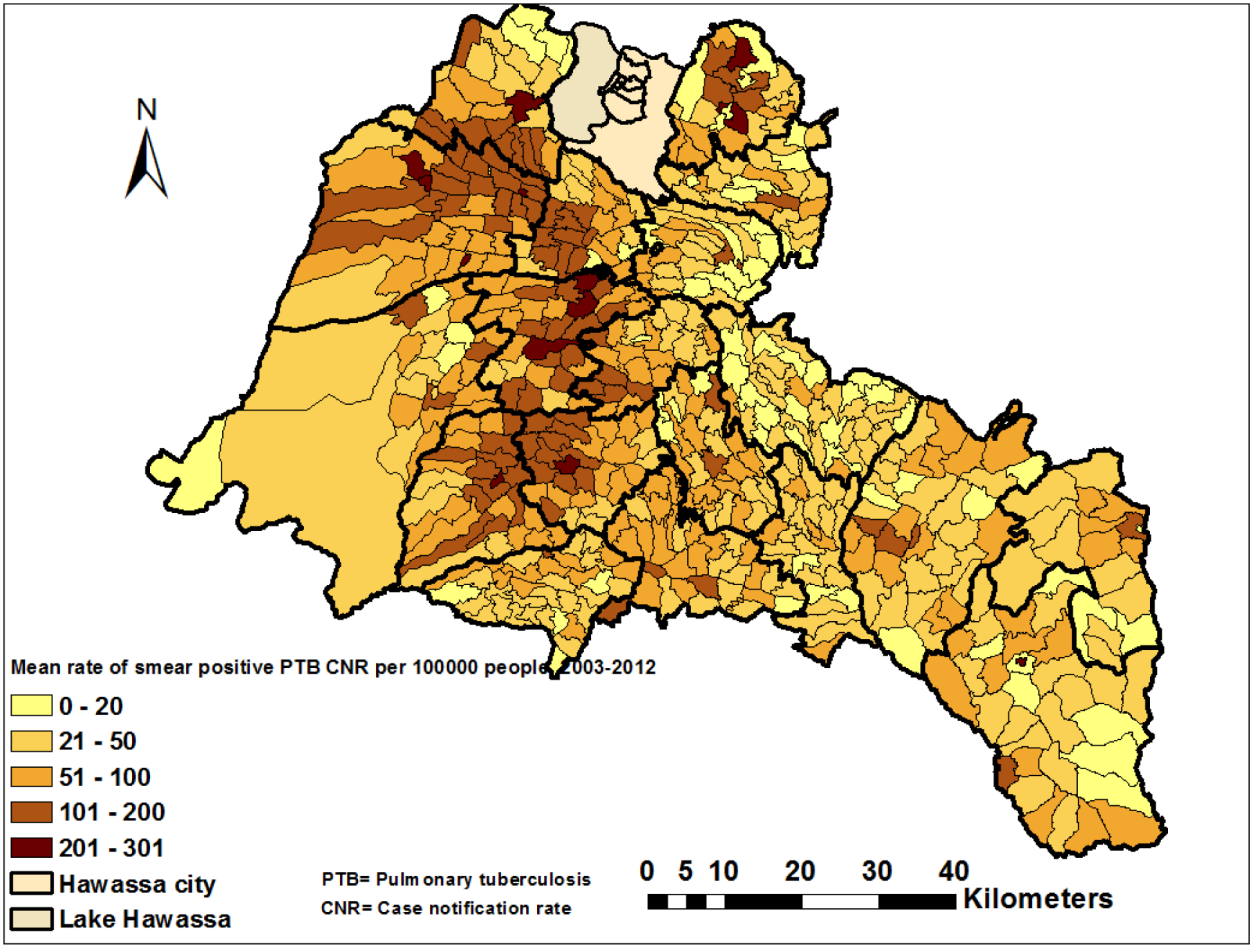
Spatial distribution of smear-positive pulmonary tuberculosis by kebele (the smallest administrative unit) in the Sidama Zone in southern Ethiopia, 2003–2012.

**Fig 4:**
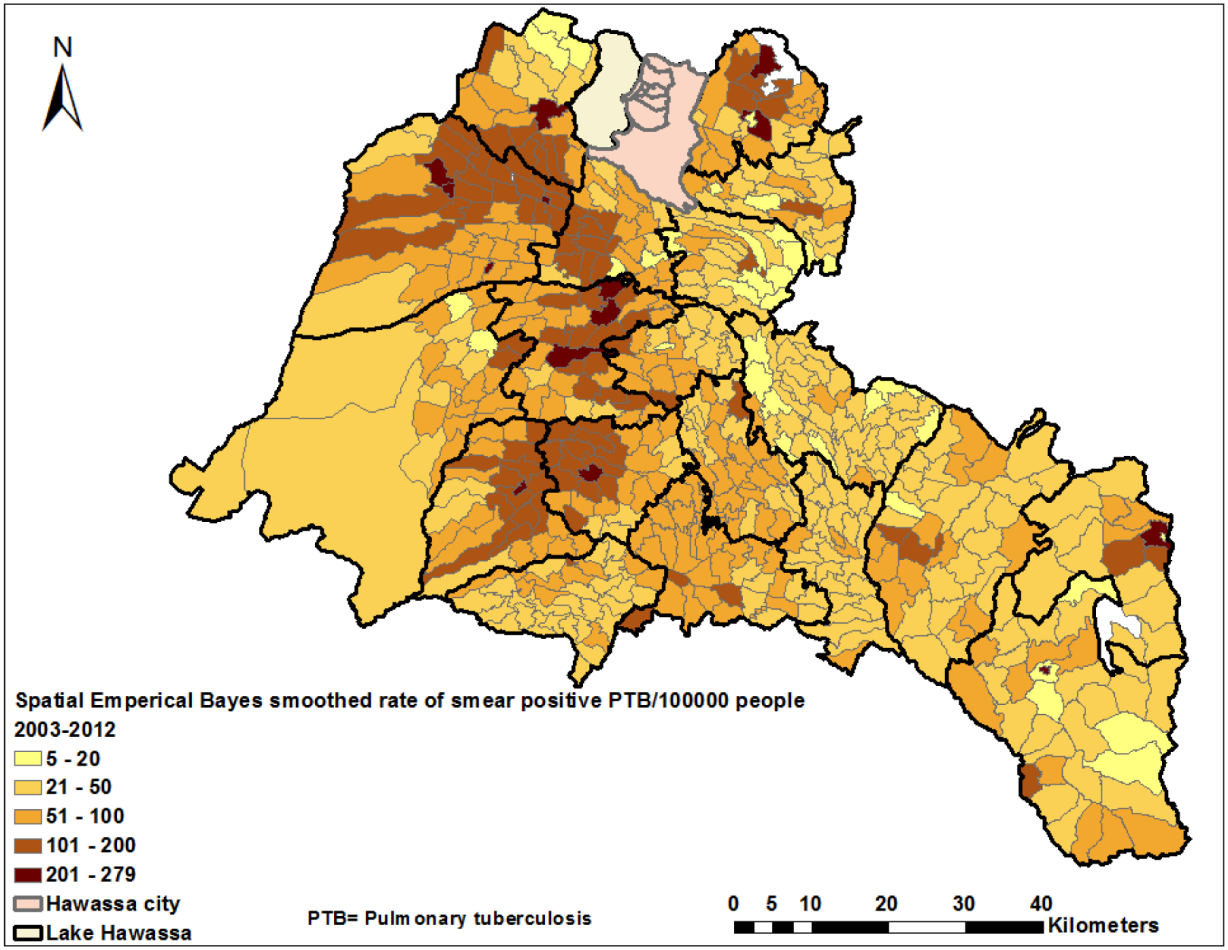
Spatial Empirical Bayes smoothed rate of smear-positive pulmonary tuberculosis in the Sidama Zone in southern Ethiopia, 2003–2012.

The CNRs varied by district across the years (S1 Fig and Table 1), with the highest CNRs being reported from two towns. The mean CNR of the study area was 76 per 100,000 people, ranging from 31 to 210 per 100,000 people in the districts (Fig 2). The CNRs in 2011 were the highest ever in all districts (ranging from 76 to 252 per 100,000 people) (Table 2). The CNRs increased from 47 in 2003 to 110 in 2012 per 100,000 for women and 63 to 118 for men (S1 Table). We observed notable variations in CNRs within districts when the data were further analyzed at the kebele level (Fig 3 and S2 Fig). High CNRs of smear-positive PTB were observed in urban kebeles, areas with a high population density and areas close to towns. There were also areas with high rates of the disease (more than 100 cases per 100,000 people), which had a population density of less than 1,000 people per square kilometer (KM^2^), while other areas had lower CNRs (less than 100 per 100,000 people) with a population density of over 1,000 per KM^2^ (S3 Fig). The mean CNRs (unsmoothed) at the kebele level over 10 years ranged from three to 263 in rural- and eight to 301 per 100,000 in urban kebeles, which was higher than the mean CNRs observed in the districts (Figs 2 and 3). Additionally, the smoothed mean CNRs ranged from five to 279 per 100,000 people during the study period (Fig 4).

**Table 2:** Case notification rates of smear-positive pulmonary tuberculosis per 100,000 people by districts in the Sidama Zone in southern Ethiopia, 2003–2012.

District Name	2003	2004	2005	2006	2007	2008	2009	2010	2011	2012	Mean CNR rate	Population Density (2012)
Shebedino	89	69	45	50	64	71	74	61	131	128	78	791
Hawassa Zuriya	61	74	55	67	66	102	55	77	118	94	76	583
Arbegona	7	6	5	1	9	46	62	33	89	57	31	519
Dale	94	71	74	50	105	118	126	103	172	159	103	1,093
Aleta wondo	53	93	83	86	89	105	101	81	132	122	90	623
Dara	9	24	82	44	28	75	32	67	76	57	50	334
Hula	49	44	67	46	62	55	61	95	108	111	70	468
Bensa	27	40	61	35	43	55	52	55	85	84	54	395
Aroresa	4	22	38	23	72	114	46	59	81	130	58	288
Boricha	122	123	74	86	85	157	98	59	151	102	106	478
Gorche	25	15	0	1	8	21	37	36	113	77	33	671
Malga	32	46	33	30	30	47	35	33	79	75	44	550
Wonsho	54	37	45	38	49	33	28	40	152	136	61	499
Loka Abaya	58	36	31	29	24	65	49	44	180	110	62	630
Chire	0	7	15	26	27	40	74	43	101	229	55	954
Bursa	41	30	52	44	75	52	71	55	90	91	60	124
Chuko	94	101	79	80	70	101	112	88	126	117	97	630
Bona	23	15	60	41	7	36	44	71	115	80	50	596
Wondo Genet	75	135	86	65	90	111	108	88	225	208	119	549
Towns												
Aleta wondo town	57	366	294	263	235	207	197	204	105	140	207	5,326
Yirgalem town	249	231	185	212	108	207	246	230	252	180	210	2,808
Sidama Zone	55	62	58	51	58	82	73	67	122	111	76	483

CNR = case notification rate

### Spatial autocorrelation analysis and spatial clustering of smear-positive PTB in the Sidama Zone

**Fig 5:**
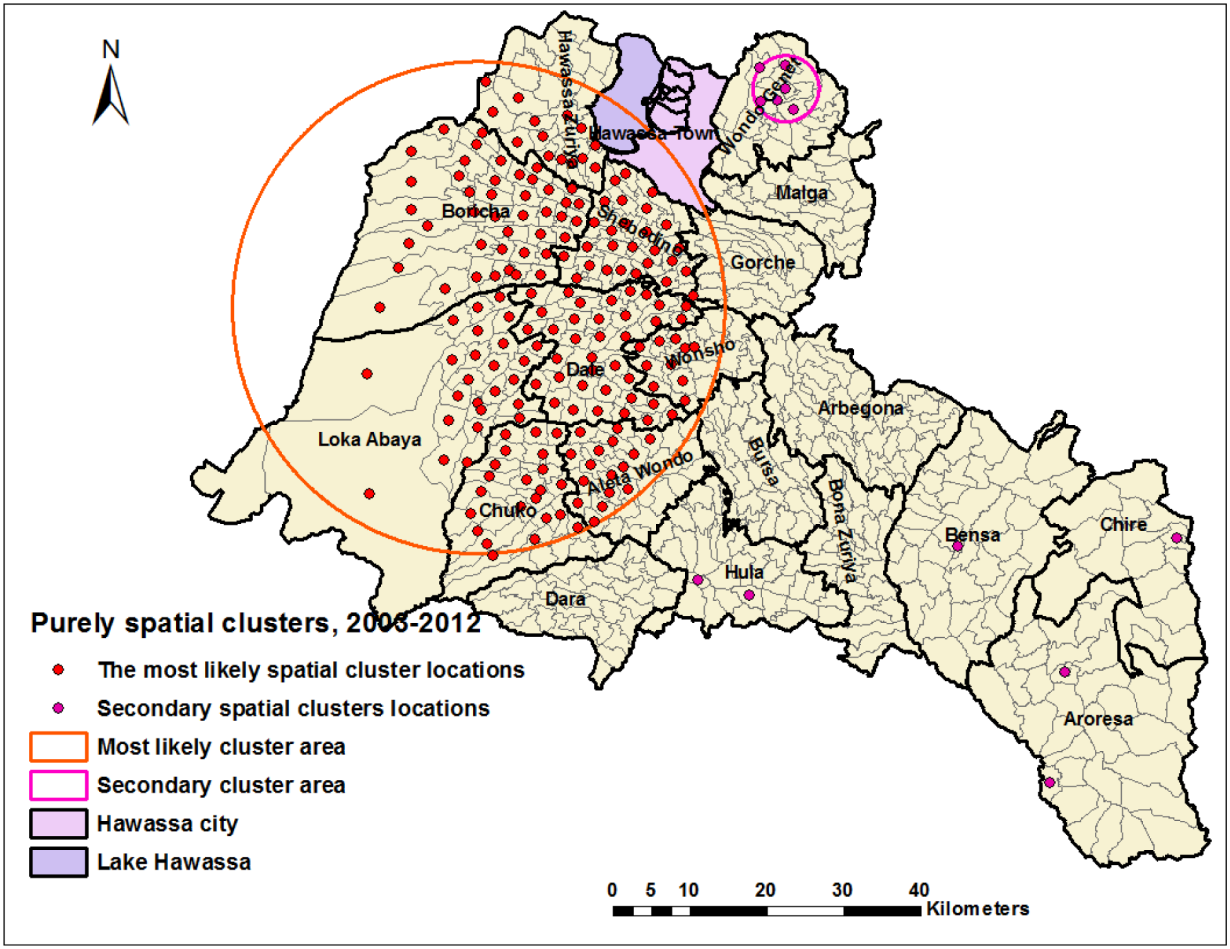
Purely spatial clusters of smear-positive pulmonary tuberculosis in the Sidama Zone in southern Ethiopia, 2003–2012.

The Global Moran’s *I* autocorrelation analysis showed that smear-positive PTB was significantly auto-correlated for each year (Table 3). In a purely spatial analysis, we identified a significant most likely cluster for a high occurrence of smear-positive PTB, which consisted of 192 locations in eight districts (Fig 5 and Table 4). The overall RR of the cluster was 2, with an observed number of 12,155 cases notified during 2003–2012, compared with 8,668 expected cases. We found secondary clusters of smear-positive PTB in the Wondo Genet, Aroresa, Hula, Chire and Bensa districts during 2003–2012, and all locations were in urban settings (Fig 5). The districts where the most likely cluster was identified accounted for 60% of cases reported during 2003–2012.

**Table 3:** Global spatial autocorrelation analyses for smear-positive pulmonary tuberculosis rate in the Sidama Zone in southern Ethiopia, 2003–2012.

Year	Moran’s *I*	Z-score	P-value	Pattern
2003	0.233391	38.4	<0.001	Clustered
2004	0.183604	30.2	<0.001	Clustered
2005	0.166729	27.4	<0.001	Clustered
2006	0.207490	27.2	<0.001	Clustered
2007	0.087827	14.8	<0.001	Clustered
2008	0.149554	24.8	<0.001	Clustered
2009	0.066854	11.2	<0.001	Clustered
2010	0.063024	10.5	<0.001	Clustered
2011	0.077268	13.1	<0.001	Clustered
2012	0.063749	10.7	<0.001	Clustered

**Table 4:** Most likely and secondary spatial clusters of smear-positive pulmonary tuberculosis cases detected by purely spatial analysis in the Sidama Zone in southern Ethiopia, 2003–2012.

Cluster	Year	Number of cluster locations	Observed cases	Expected cases	Likelihood ratio	Relative risk	P-value
Most likely cluster	2003–2012	192	12,155	8,668	1134	2	<0.001
Secondary	2003–2012	8	889	400	225	2.3	<0.001
2^nd^ secondary	2003–2012	1	201	85	56.9	2.37	<0.001
3^rd^ secondary	2003–2012	1	112	38	46.75	2.94	<0.001
4^th^ secondary	2003–2012	1	118	53	29.5	2.23	<0.001
5^th^ secondary	2003–2012	1	82	30	28.8	2.65	<0.001
6^th^ secondary	2003–2012	1	64	23	26.5	2.92	<0.001
7^th^ secondary	2003–2012	1	50	24	11	2.1	0.013

**Fig 6:**
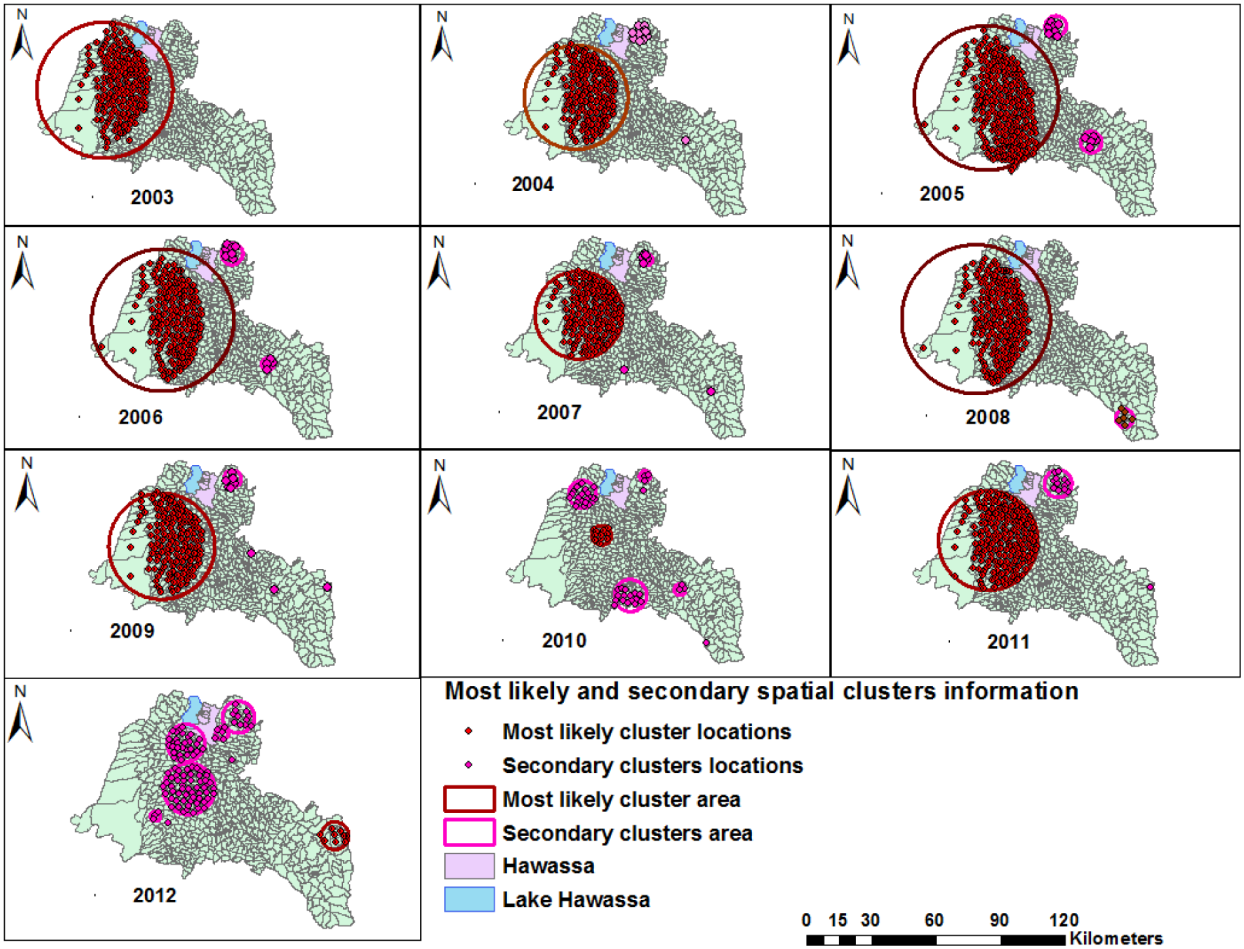
Purely spatial clusters of smear-positive pulmonary tuberculosis by year in the Sidama Zone in southern Ethiopia, 2003–2012.

**Fig 7:**
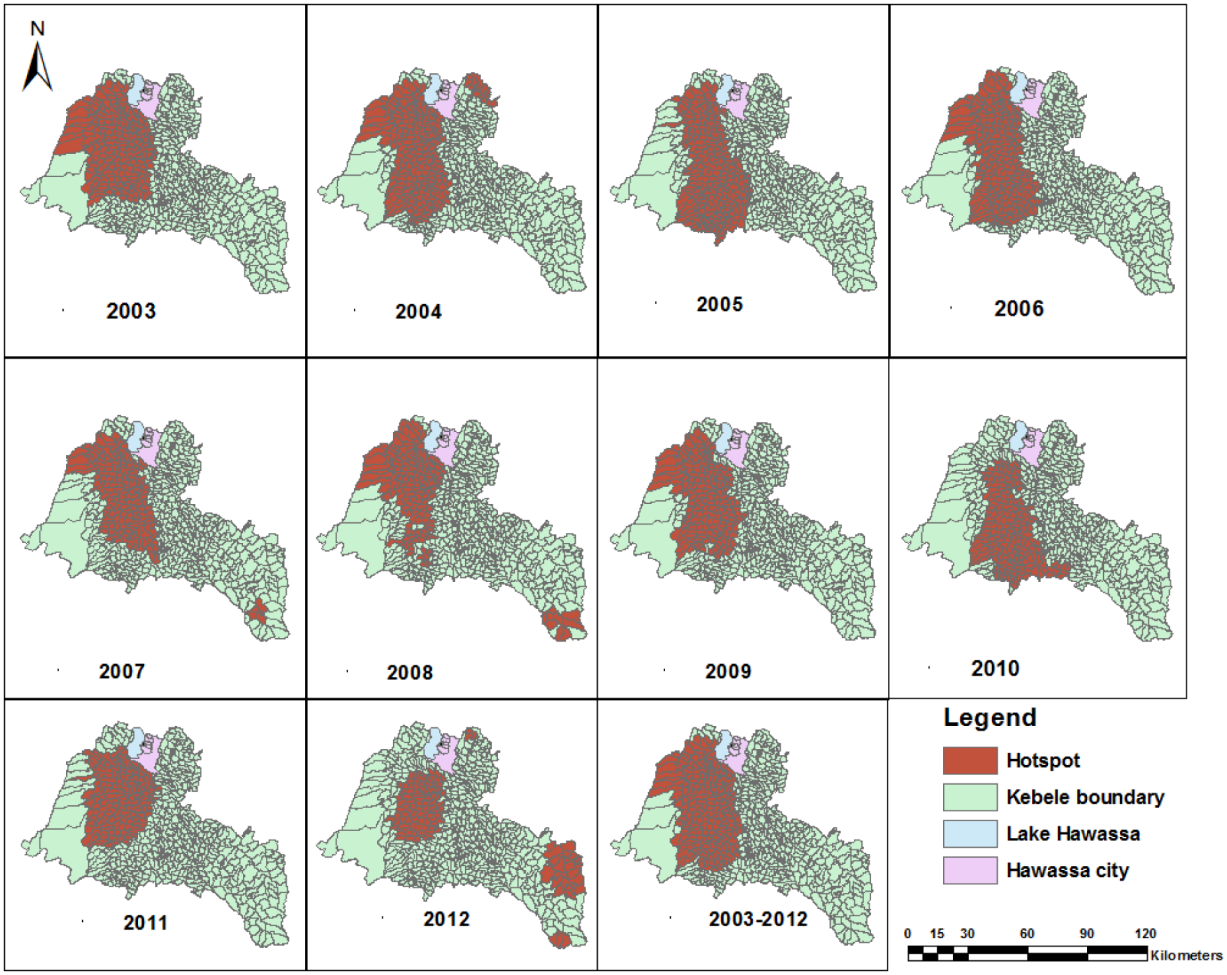
Significant clusters of high rates of smear-positive pulmonary tuberculosis identified by Gi* statistics in the Sidama Zone in southern Ethiopia, 2003–2012.

We observed for the pattern and stability of spatial clusters in each year during the study period, and the clusters were stable in most districts except in 2010 and 2012 (Fig 6). The clusters were detected in the Shebedino, Dale, Aleta wondo, Dara, Hula, Boricha, Hawassa Zuriya, Wonsho, Loka Abaya and the Chuko districts (from 2003–2009 and 2011), the Dale district (in 2010) and the Chire district (the southeastern border of the study area) in 2012. Furthermore, the most likely clusters were accompanied by secondary clusters during the study period, and the secondary spatial clusters were detected in all years except in 2003 (Fig 6 and Table 5). The Gi* statistic also identified local clusters of smear-positive PTB in the same areas identified by scan statistics, except for differences in a few locations (Fig 7).

**Table 5:** Purely spatial clusters of smear-positive pulmonary tuberculosis with significant most likely and secondary clusters in the Sidama Zone in southern Ethiopia, 2003–2012.

Cluster type	Year	Number of cluster locations	Observed cases	Expected cases	Likelihood ratio	Relative risk	P-value
Most likely cluster	2003	176	909	475	291	3.89	<0.001
Most likely cluster	2004	155	891	482	232	3.1	<0.001
Secondary	2004	12	141	47	64	3.2	<0.001
2^nd^ secondary	2004	1	24	6	16	4.2	<0.001
Most likely cluster	2005	235	958	637	147	2.6	<0.001
Secondary	2005	9	74	27	27	2.8	<0.001
2^nd^ secondary	2005	5	57	19	25	3.1	<0.001
Most likely	2006	178	817	483	169	2.7	<0.001
Secondary cluster	2006	9	69	26	24	2.7	<0.001
2^nd^ secondary	2006	4	39	16	11	2.5	0.003
Most likely	2007	152	822	509	128	2.2	<0.001
Secondary	2007	1	22	2	36	13.5	<0.001
2^nd^ Secondary	2007	5	55	18	24	3.1	<0.001
3^rd^ Secondary	2007	2	22	4	21	6.3	<0.001
Most likely cluster	2008	192	1,416	926	204	2.3	<0.001
Secondary cluster	2008	6	52	26	9	2	0.032
Most likely cluster	2009	155	1,139	697	190	2.3	<0.001
Secondary clusters	2009	8	111	50	28	2.3	<0.001
2^nd^ Secondary	2009	1	16	4	11	4.2	0.011
3^rd^ secondary	2009	1	25	9	10	3	0.014
4^th^ secondary	2009	1	18	5	10	3.6	0.020
Most likely cluster	2010	11	187	63	83	3.2	<0.001
Secondary cluster	2010	20	146	83	20	1.8	<0.001
2^nd^ Secondary	2010	1	24	5	18	4.7	<0.001
3^rd^ Secondary	2010	16	90	46	17	2.04	<0.001
4^th^ secondary	2010	3	47	20	13	2.4	<0.001
5^th^ Secondary	2010	3	36	14	12	2.7	0.002
6^th^ secondary	2010	1	14	3	11	4.7	0.009
Most likely cluster	2011	180	2,019	1,531	122	1.6	<0.001
Secondary cluster	2011	13	187	95	35	2.02	<0.001
2^nd^ Secondary	2011	1	38	7	33	5.5	<0.001
Most likely cluster	2012	9	205	65	100	3.3	<0.001
Secondary cluster	2012	13	196	91	46	2.2	<0.001
2^nd^ Secondary	2012	56	658	468	39	1.5	<0.001
3^rd^ Secondary	2012	29	359	250	22	1.5	<0.001
4^th^ Secondary	2012	3	42	17	13	2.6	<0.001
5^th^ Secondary	2012	4	73	39	12	1.9	0.003
6^th^ Secondary	2012	1	25	8	11	3.1	0.006
7^th^ Secondary	2012	1	17	5	9	3.5	0.05

### Space-time clustering

**Fig 8:**
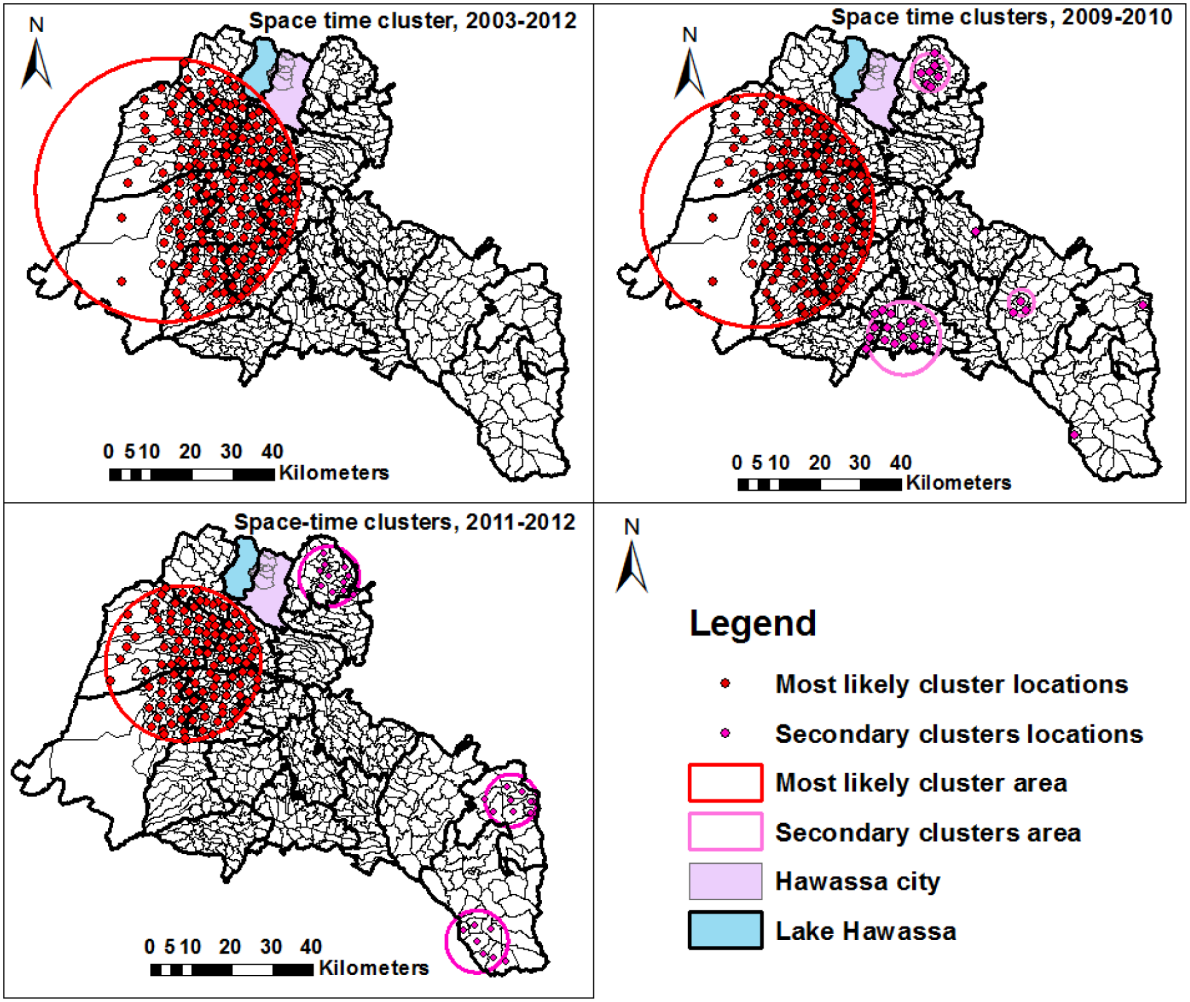
Significant space-time clusters of smear-positive pulmonary tuberculosis in the Sidama Zone in southern Ethiopia, 2003–2012, 2009–2010, and 2011–2012.

In a space-time cluster analysis of smear-positive PTB during 2003–2012, we found the most likely clusters at 193 locations in eight districts (RR = 1.92, p<0.001) with 7,584 observed and 4,738 expected cases (Fig 8 and Table 6). The locations for space-time clusters were the same with the locations in which the purely spatial clusters were detected, except for the secondary clusters. We looked into the pattern of recent space-time clusters in sub-time- phases from 2009–2010 and 2011–2012 and from 2009–2010 we identified the most likely space-time cluster in 154 locations (RR = 1.93, P<0.001), with six secondary clusters in 29 locations. Lastly, in 2011–2012, the most likely cluster was identified in 113 locations (RR = 1.6), with three secondary clusters in 27 locations (Fig 8 and Table 6).

**Table 6:** Space-time clusters of smear-positive pulmonary tuberculosis in the Sidama Zone in southern Ethiopia, 2003–2012.

Clusters	Data Year	Time frame	Number of cluster locations	Observed cases	Expected cases	Likelihood ratio	Relative risk	P-value
Most likely cluster	2003–2012	2008–2012	193	7,584	4,738	974	1.92	<0.001
Most likely cluster	2009–2010	2009	154	1,139	677	162	1.93	<0.001
Secondary cluster	2009–2010	2009	7	111	49	30	2.33	<0.001
2^nd^ secondary	2009–2010	2010	16	90	47	16	1.95	<0.001
3^rd^ secondary	2009–2010	2010	3	47	21	13	2.33	0.003
4^th^ secondary	2009–2010	2009	1	16	4	11	4.31	0.014
5^th^ secondary	2009–2010	2009	1	18	5	11	3.74	0.025
6^th^ secondary	2009–2010	2010	1	14	3	10	4.60	0.028
Most likely cluster	2011–2012	2011	113	1,330	878	115	1.6	<0.001
Secondary	2011–2012	2012	9	205	66	94	3.14	<0.001
2nd secondary	2011–2012	2012	12	196	94	43	2.12	<0.001
3rd secondary	2011–2012	2012	7	91	45	18	2.03	<0.001

## Discussion

We found spatial and spatio-temporal clusters and variations in the distribution of smear-positive PTB in the northwestern and east central districts of the study area. These clusters were stable over the years with the exception of a few location differences. This could be explained by a high transmission over many years due to the existence of disproportionate high-risk factors, and a varying program performance.

In our finding, most cluster locations were identified in urban areas, rural areas with a high population density, as well as neighboring areas close to towns and areas near road networks, which connect major towns. Various studies have reported that poor socioeconomic conditions such as social inequality, low income, poverty, poor housing conditions, overcrowding and social unrest could all be risk factors for the high burden and variations of disease occurrence [2, 3, 5, 6, 15, 20, 30–33]. In addition, patient care factors [34] and poor access to health care and TB control services could also contribute to a high rate of the disease [35] since infectious cases may remain undiagnosed and may not acquire treatment, which could consequently contribute to the transmission dynamics of the disease. Furthermore, most urban kebeles where the clusters identified were the capitals of the districts, had market places, had public transportation routes and were the hub for different socio-economic activities. The better access to road and movement using public transportation in a crowded and poorly ventilated environment may assist in facilitating contact with infectious cases, which could favor a transmission of the disease in the locations where clusters were detected [36–38]. Moreover, TB can be associated with HIV and other risk factors such as a decreased immunity [39, 40]; however, in our data we could not compare the clustering of smear-positive TB with the geographic distribution of HIV prevalence because the data on spatial distribution of HIV were not available. Therefore, the inclusion of these factors in cluster analyses in the future may help to improve our understanding of their effect on the clusters of the disease in the study area.

The space-time statistic also identified clustering in the same locations detected by the purely spatial scan statistics and Gi* statistics. Our finding was in agreement with other studies that report the existence of both spatial and space-time clusters in the same areas, which could support the evidence of an uneven distribution and burden of the disease [8, 9, 13, 15]. Likewise, both methods of the local analysis of disease clustering (Kulldroff’s scan statistics and Gi* statistics) have identified unusually high rates of smear-positive PTB in the same districts, with the exception of a few differences in the number of locations. Therefore, the methods (spatial, space-time and Gi* statistics) can be useful and robust tools to identify and detect areas of unusually high disease occurrences. Moreover, cluster analysis and mapping of the disease distribution could add value beyond that which can be obtained by presenting the disease rates in a table, as a cluster analysis helps identify areas with unusually high disease rates, which have less likely occurred by chance [7].

A study from Mexico reported that improving TB control efforts could help reduce the transmission and change the geographic distribution of TB [17]. In our study, despite different intervention programs aimed at reducing disease transmission and improving case detection over many years, the unusually high rates of the disease persisted in the same places, with the most likely spatial clusters showing a stable pattern in the preceding years during the study, except in 2010 and 2012. This could explain, at least in part, that the interventions may not be properly focused on influencing the disease epidemiology or could be due to a low case finding and poor treatment of infectious cases, which may indicate the continued transmission of the disease.

In 2008, a new secondary spatial cluster was identified along the southwestern border of the study area. This area is in an urban setting with neighboring kebeles and the area has a marketplace, better road and public transportation access, a high population density and a lower altitude compared with other areas in the southwest, which could contribute to a higher CNR. In 2010, there was a change in the distribution pattern of the clusters. This could be because of an intensive case finding campaign conducted in nine districts in the study area, which could contribute to the observed change in the distribution pattern of the disease. Since 2011, a community-based TB case finding intervention has been launched in all districts, and the intervention has improved access to TB care and increased the CNR in the study area [41]. Thus, after one year of implementation of the intervention in 2012, the most likely cluster shifted to the southeastern border (the Chire district) of the study area; nonetheless, the secondary clusters persisted in locations where the most likely clusters were detected in the preceding years. This could be due to the fact that the access to- and utilization of health services have been limited, and that the intervention increased access to the services, as reflected by the increased case finding. This implies that devising a focused intervention in the future based on the disease burden could be effective in tackling the transmission in areas where the clusters were detected.

On the other hand, an improved surveillance, and an improved access to- and utilization of TB control services may help increase TB case notification rates or could contribute to a decline in the disease transmission, which might also affect the disease notification rates. Further investigation is therefore needed to better understand the role of health service access to the case notifications and clustering of smear-positive PTB in the study area.

Conversely, the eastern-, east central- and southern parts of the study area had lower smear-positive PTB rates than the west central- and northern parts of the study area. This is possibly due to poor access to TB control services or possibly due to a lower burden of the disease. Studies have shown that environmental factors such as altitude also correlated with the incidence of tuberculosis [42, 43], and further analysis is required to help understand the factors that could contribute to the lower notification rates such as access to- and the availability of TB control services, as well as environmental- and socio-economic factors.

It has been suggested that the “one fits all” interventions could not be equally effective in different areas with variations in disease occurrence because areas with high disease rates, which are less likely to have occurred by chance, may need more attention than areas with a low risk for targeting interventions. Therefore, our findings suggest that policymakers and health authorities should better understand and prioritize areas that need attention for focused interventions, as well as for strengthening TB surveillance.

The limitations of our study were that the study was not a population-based survey, instead based on surveillance data of smear-positive PTB cohorts. Hence, cases that were not diagnosed, or with a delayed diagnosis and smear negative but culture positive cases that were not captured by sputum-smear microscopy, could be missed and underestimate the CNRs. Cases may not be registered after being diagnosed; however, the possibility of missing was rare because it is mandatory for health facilities to link TB cases to DOTS services and to report to the next administrative levels once the cases are diagnosed and the treatment is free of charge.

The strengths of our study were that the study was based on true CNRs since cases were linked to their home address; in addition, cases that were registered outside the study area (nearby areas) were also linked to their actual address in the study area. We used the kebele (small scale) as a unit of analysis and included urban and rural settings, which helped in improving our understanding of the spatial epidemiology of the disease in a wider geographic context. The long study period (10 years) enabled us to assess the spatial pattern and stability of clusters of the disease in the study area. Lastly, missing information in our data for the unit of analysis was only 3%, so the percentage was too small to affect our results.

## Conclusion

We found spatio-temporal clusters and spatial variations of smear-positive PTB in the Sidama Zone. As a result, TB in the study area did not uniformly occur in different geographic settings, and exhibited a non-random distribution. The findings can be used to guide TB control programs to help devise effective TB control strategies for the geographic areas characterized by the highest CNRs. Further investigations based on individual level locations are needed to identify the presence of localized spatial clustering and causes for unusually high rates in those areas by incorporating socioeconomic factors, type of TB strain and access to health services so as to improve our understanding of the possible causes for unusually high disease rates.

## Supporting Information

S1 Fig Distribution of smear-positive pulmonary tuberculosis by district in the Sidama Zone in southern Ethiopia, 2003–2012. (TIF)

S2 Fig Distribution of smear-positive pulmonary tuberculosis by kebele (the smallest administrative unit) in the Sidama Zone in southern Ethiopia, 2003–2012. (TIF)

S3 Fig Distribution of population density in 2012 and mean case notification rates of smear-positive pulmonary tuberculosis in the Sidama Zone in southern Ethiopia, 2003–2012. (TIF)

S1 Table Trends of smear-positive pulmonary tuberculosis case notifications in the Sidama Zone in southern Ethiopia, 2003–2012. (DOCX)

S2 Table Data collection tools. (XLSX)
