# Supplementary figures and images for: Spatio-Temporal Analysis of Smear-Positive Tuberculosis in the Sidama Zone, Southern Ethiopia
Source: PLoS One. 2015 Jun 1;10(6):e0126369. doi: 10.1371/journal.pone.0126369 (PMC4451210; doi:10.1371/journal.pone.0126369)

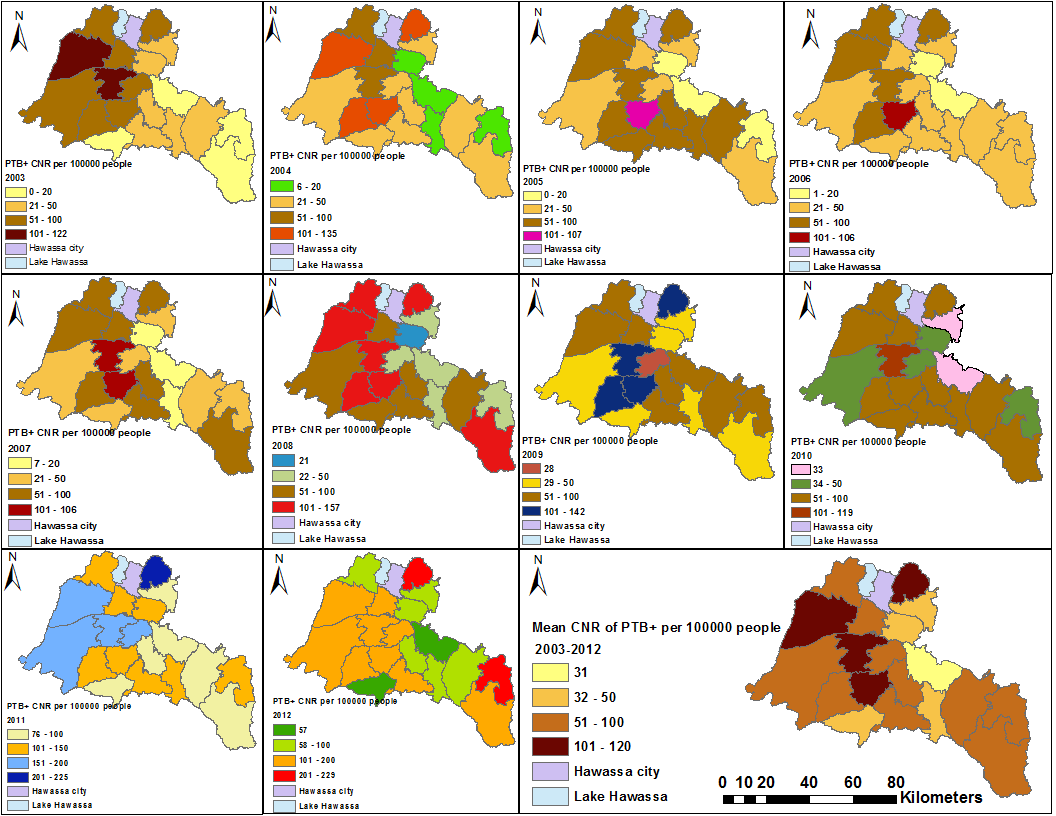

Supplement: S1 Fig — (TIF) [file pone.0126369.s001.tif]

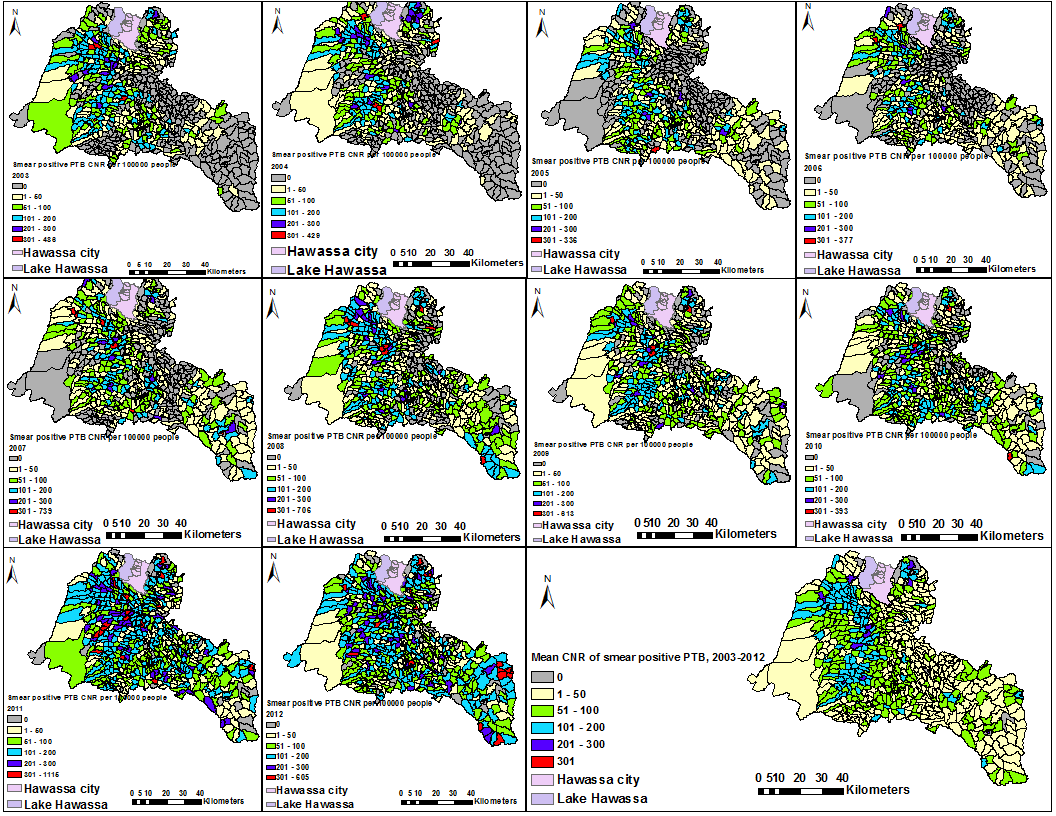

Supplement: S2 Fig — (TIF) [file pone.0126369.s002.tif]

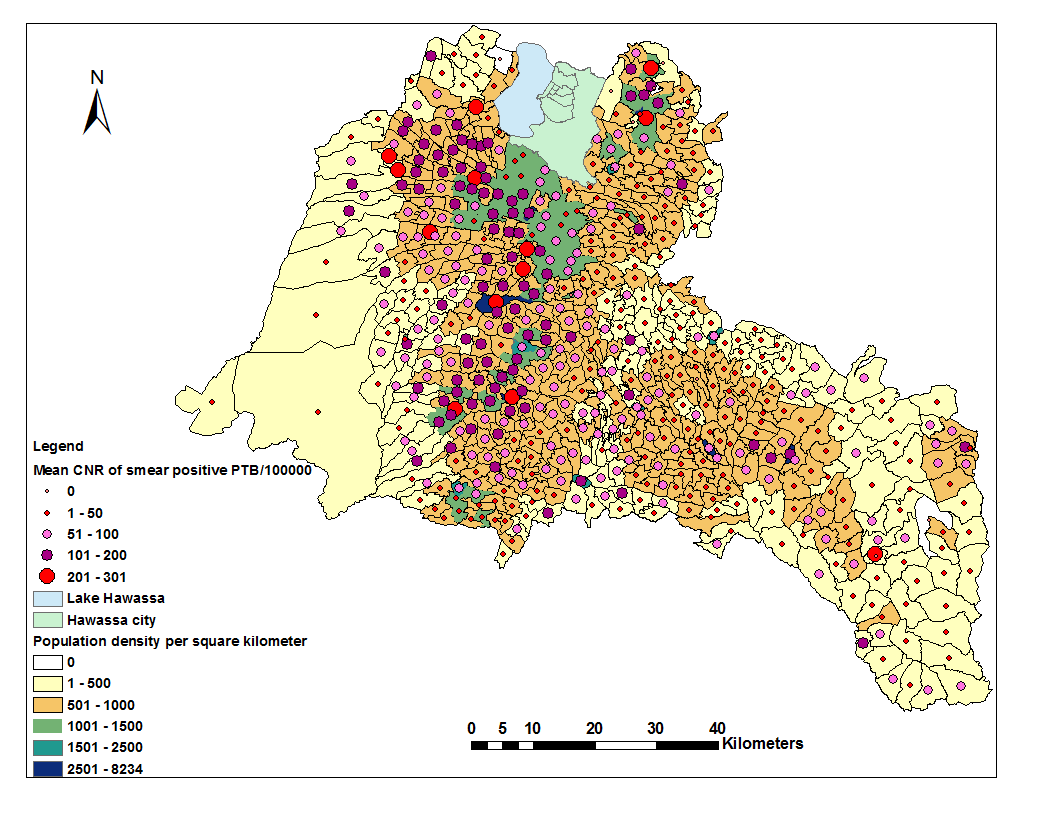

Supplement: S3 Fig — (TIF) [file pone.0126369.s003.tif]
