# Supplementary material for: Spatio-Temporal Analysis of Smear-Positive Tuberculosis in the Sidama Zone, Southern Ethiopia
Source: PLoS One. 2015 Jun 1;10(6):e0126369. doi: 10.1371/journal.pone.0126369 (PMC4451210; doi:10.1371/journal.pone.0126369)
Supplement: S1 Table — (DOCX) [file pone.0126369.s004.docx]

Table S1: Trends of smear positive case notifications in the Sidama Zone in southern Ethiopia, 2003-2012

| Characteristics | 2003  N (%) | 2004  N (%) | 2005  N (%) | 2006  N (%) | 2007  N (%) | 2008  N (%) | 2009  N (%) | 2010  N (%) | 2011  (N %) | 2012  N (%) |
| --- | --- | --- | --- | --- | --- | --- | --- | --- | --- | --- |
| Total cases | 1,358 | 1,586 | 1,574 | 1,434 | 1,734 | 2,502 | 2,286 | 2,156 | 4,056 | 3,851 |
| Sex |  |  |  |  |  |  |  |  |  |  |
| Male | 785 (58) | 886 (56) | 856 (54) | 794 (55) | 943 (54) | 1339 (54) | 1295 (57) | 1215 (56) | 2105 (52) | 2017 (52) |
| Female | 573 (42) | 700 (44) | 718 (46) | 639 (45) | 791 (46) | 1158 (46) | 991 (43) | 938 (44) | 1951 (48) | 1834 (48) |
| Residence |  |  |  |  |  |  |  |  |  |  |
| Urban | 156 (12) | 261 (16) | 201 (13) | 218 (15) | 217 (12) | 266 (11) | 308 (13) | 252 (12) | 305 (8) | 264 (7) |
| Rural | 1202 (88) | 1325 (84) | 1373 (87) | 1216 (85) | 1517 (88) | 2236 (89) | 1978 (87) | 1904 (88) | 3751 (92) | 3587 (93) |
| Age category |  |  |  |  |  |  |  |  |  |  |
| 0-14 | 132 (10) | 153 (10) | 149 (10) | 145 (10) | 154 (9) | 209 (9) | 184 (8) | 178 (8) | 373 (9) | 279 (7) |
| 15-24 | 480 (36) | 520 (33) | 524 (34) | 472 (34) | 582 (34) | 771 (31) | 765 (34) | 720 (34) | 1164 (29) | 1018 (27) |
| 25-34 | 428 (32) | 495 (31) | 509 (33) | 437 (31) | 537 (31) | 801 (32) | 683 (30) | 667 (31) | 1234 (30) | 1170 (30) |
| 35-44 | 153 (11) | 206 (13) | 197 (13) | 169 (12) | 236 (14) | 317 (13) | 295 (13) | 248 (12) | 572 (14) | 618 (16) |
| 45-54 | 82 (6) | 126 (8) | 110 (7) | 102 (7) | 107 (6) | 212 (9) | 205 (9) | 183 (9) | 426 (11) | 451 (12) |
| 55-64 | 43 (3) | 49 (3) | 49 (3) | 47 (3) | 57 (3) | 110 (4) | 96 (4) | 91 (4) | 184 (5) | 195 (5) |
| 65+ | 28 (2) | 31 (2) | 28 (2) | 35 (3) | 35 (2) | 52 (2) | 39 (2) | 61 (3) | 101 (3) | 115 (3) |
| CNR/100,000 people |  |  |  |  |  |  |  |  |  |  |
| Sex |  |  |  |  |  |  |  |  |  |  |
| Men | 63 | 68 | 63 | 56 | 63 | 87 | 82 | 74 | 125 | 118 |
| Women | 47 | 55 | 54 | 46 | 54 | 76 | 64 | 58 | 118 | 110 |
| Residence |  |  |  |  |  |  |  |  |  |  |
| Urban | 121 | 191 | 138 | 141 | 128 | 155 | 173 | 136 | 162 | 126 |
| Rural | 52 | 54 | 54 | 46 | 55 | 78 | 68 | 63 | 121 | 110 |
| Age category |  |  |  |  |  |  |  |  |  |  |
| 0-14 | 11 | 12 | 11 | 10 | 10 | 14 | 12 | 11 | 22 | 16 |
| 15-24 | 106 | 110 | 105 | 90 | 106 | 136 | 132 | 120 | 189 | 159 |
| 25-34 | 132 | 145 | 142 | 117 | 136 | 198 | 164 | 154 | 280 | 254 |
| 35-44 | 79 | 101 | 92 | 76 | 101 | 131 | 119 | 96 | 218 | 225 |
| 45-54 | 72 | 105 | 88 | 78 | 78 | 150 | 140 | 121 | 274 | 280 |
| 55-64 | 70 | 76 | 73 | 67 | 77 | 144 | 122 | 113 | 221 | 225 |
| 65+ | 47 | 49 | 42 | 51 | 48 | 70 | 51 | 77 | 124 | 135 |
| All | 55 | 62 | 58 | 51 | 58 | 82 | 73 | 66 | 122 | 111 |

CNR= Case notification rate
